# Accessing and utilizing clinical and genomic data from an electronic health record data warehouse

**DOI:** 10.1186/s41231-023-00140-0

**Published:** 2023-03-02

**Authors:** Cosby G. Arnold, Brandon Sonn, Frederick J. Meyers, Alexis Vest, Richie Puls, Estelle Zirkler, Michelle Edelmann, Ian M. Brooks, Andrew A. Monte

**Affiliations:** 1 Department of Emergency Medicine, School of Medicine, University of California, Davis, 4150 V Street #2100, Sacramento, CA 95817, USA; 2 Department of Emergency Medicine, University of Colorado Denver-Anschutz Medical Center, University of Colorado School of Medicine, Mail Stop B-215, 12401 East 17th Avenue, Aurora, CO 80045, USA; 3 Department of Internal Medicine, University of California, Davis, School of Medicine, 4150 V Street #3100, Sacramento, CA 95817, USA; 4 Department of Biomedical Informatics, University of Colorado School of Medicine, Anschutz Health Sciences Building, 1890 N. Revere Court, Mailstop F600, Aurora, CO 80045, USA; 5 Rocky Mountain Poison & Drug Center, Denver Health and Hospital Authority, 1391 Speer Blvd Unit 600, Denver, CO 80204, USA

**Keywords:** Electronic health record, Data warehouse, Big data, Genomics

## Abstract

Electronic health records (EHRs) and linked biobanks have tremendous potential to advance biomedical research and ultimately improve the health of future generations. Repurposing EHR data for research is not without challenges, however. In this paper, we describe the processes and considerations necessary to successfully access and utilize a data warehouse for research. Although imperfect, data warehouses are a powerful tool for harnessing a large amount of data to phenotype disease. They will have increasing relevance and applications in clinical research with growing sophistication in processes for EHR data abstraction, biobank integration, and cross-institutional linkage.

## Background

The widespread adoption of electronic health records (EHRs) affords an unprecedented opportunity to leverage clinical data for research. EHRs contain a large amount of clinical information that, when combined with other data sources such as ‘omics, enhances the potential for research discovery. The secondary use of EHR data does not involve subject recruitment or prospective data collection. It often qualifies for expedited rather than full institutional review board (IRB) review and may qualify for exemption if the data is deidentified. Vast reservoirs of such patient data derived from clinical encounters promises to accelerate patient-centered research and advance medical discoveries at decreased cost [1].

Data warehouses can store enormous quantities of structured, semi-structured, and unstructured data extracted from EHRs and other sources. When combined with other modalities such as images, claims data, public health outcome data, prescription data, radiology, lab databases, and wearables [2], these data are called a Health Data Warehouse, or Clinical Data Warehouse. The cloud-based clinical data warehouse at the University of Colorado is called Health Data Compass (HDC), and has been described elsewhere [3].

Health data warehouses increasingly support the storage and re-use of large quantities of clinically-derived electronic data for clinical and translational research, clinical operations, and quality improvement. These data are longitudinal and amenable to descriptive analytics or trend analysis to evaluate patient outcomes over time in a longitudinal patient record. Secondary use of well curated EHR data can be relatively inexpensive and efficient, and includes a vast amount of clinical detail not available from administrative claims data [4, 5]. EHR data can be linked to additional data sources and provide a detailed and longitudinal approach to understanding health across the lifecycle.

There are some caveats to these approaches, however. For example, to protect patient privacy these data necessarily have tightly restricted access controls. In addition, healthcare data can be difficult to analyze until carefully validated and transformed into analytic datasets. Healthcare data are also vast and heterogenous, requiring large data storage and delivery platforms to manage which come with their own complexities and expenses. Finally, healthcare data are famously non-interoperable and harmonizing with data from outside the original health system can pose significant challenges.

Despite the many advantages of EHR data for research, knowing how to access and use these resources is often not intuitive and can lead to frustrations as researchers may require repeated attempts to get the data necessary to answer the desired research questions. The objective of this paper is to describe the opportunities, challenges, and mechanics of using a health data warehouse for clinical research. We explain the types of data available, limitations of EHR data, and steps necessary to obtain the data desired in a timely fashion.

## Main text

### Overview of using EHR data for research

#### Data types and steps for data request

Data abstracted from the EHR for secondary use research falls broadly into three categories, each with specific compliance expectations. Datasets are classified as identified, limited, or de-identified. Identified data contains personal health information (PHI), limited data has partial removal of PHI, and de-identified data does not include PHI. The investigator should request the minimum amount of PHI necessary to accomplish the goals of the research.

Datasets containing PHI are necessarily managed with the greatest precautions. Identified data requires full IRB review while de-identified data often qualifies for exemption. Investigators using only limited data may obtain a waiver of consent. In addition, institutions may have specific requirements for data use not addressed by IRB regulations [6].

Prior to submitting a data request, we would strongly recommend that the investigator or their team has engaged with a statistician or analyst to accurately determine which data are likely to be needed, and to have a plan for post-abstraction cleaning and curation (e.g., one-hot-encoding of categorical variables, imputation of missing data). Knowing in advance what challenges the data may pose will often result in a faster data delivery and is excellent prep-to-research training. Once one knows which specific variables (and thus PHI) are needed, one might obtain IRB approval [7, 8]. Furthermore, fostering a team-science approach from the outset allows subject matter experts to weigh in when appropriate. For example, certain data may not be in your clinical data warehouse and thus having a clinician on hand might facilitate locating these data. Although data analysts have access to the entire EHR, the EHR format can differ from what clinicians and investigators see when referencing the same information. An initial chart review that identifies the variables of interest and where they reside in the medical record will streamline the analyst request, subsequent data validation, and delivery process. The data analyst responsible for coding will match these specific variables with those appearing within the “back end” view of the EHR data warehouse to ensure that the investigator receives the correct data.

#### Timelines and considerations for data acquisition

The timeline for data deliveries often depends upon multiple factors. Administrative factors might include other IRB approvals, the availability of research information technology (IT) services to provision a suitable environment for data storage, or availability of specialist statistical software. Informatics factors will include the speed with which the analyst can identify and retrieve the data from the warehouse, the amount and complexity of data to be pulled, the availability of the data analyst team, and verifying that the data delivery is consistent with the request.

Initial planning meetings with data analysts should aim to determine the scope of the project, including whether the project is a feasibility study, clinical research study, or dissemination and implementation study. As a first step, the investigator and analyst should use pertinent inclusion and exclusion criteria to define a specific cohort for investigation within the bounds of their approvals. Alternatively, the investigator may specify a cohort in advance and provide personal identifiers (e.g., medical record number or encounter number) as a guide for data extraction. The investigator will also specify the clinical variables requested. Being specific in this step facilitates accurate and efficient data retrieval. Although tempting for some investigators to ask for the entirety of a subject’s medical record, this approach results in unwieldy amounts of data that take longer to obtain and renders the data cleaning process excessively time consuming. Therefore, it is prudent for researchers to have spent time working with a statistician to identify what specific variables are essential to their study and only request these data.

#### After the data delivery

In many cases, and especially if receiving raw EHR data, the investigator should assess for accuracy and arrange for the data to be cleaned, and if necessary, mapped to a common data model. One method of ensuring accuracy involves verifying a subset of the data delivered with medical records by chart review. There is risk for miscommunication during the data request and retrieval process and there are multiple locations within the EHR where the desired variables might reside. Comparing the data delivered with manual chart review on a subset of the study cohort is critical to ensure delivery of the correct variables.

Data cleaning is a crucial step in using EHR data for research. EHR data are originally intended for clinical care rather than research, and there is variation in specific data points that must be recoded for statistical analyses. For example, many factors influence how a drug is displayed within the medical record and how it is subsequently delivered in the final report for medication data. Factors that can make the same medication look different within the data report include: generic versus brand name, long acting versus immediate release formulation, which department ordered the medication, the form used to record medication history, the pharmacy that filled the prescription, the unit of measure, or the route of delivery. Laboratory values may also require review because reference ranges can vary depending on laboratory location and equipment used; each source of common lab tests will result in a separate data entry. For instance, sodium from a point of care machine, blood, and urine will all have their own separate discrete entries in the EHR. In fact, there are 146 different sodium lab values with distinct providence and structure in our local EHR. Although arduous, data cleaning is essential to ensure the integrity of the data prior to performing any analyses.

**Fig. 1:**
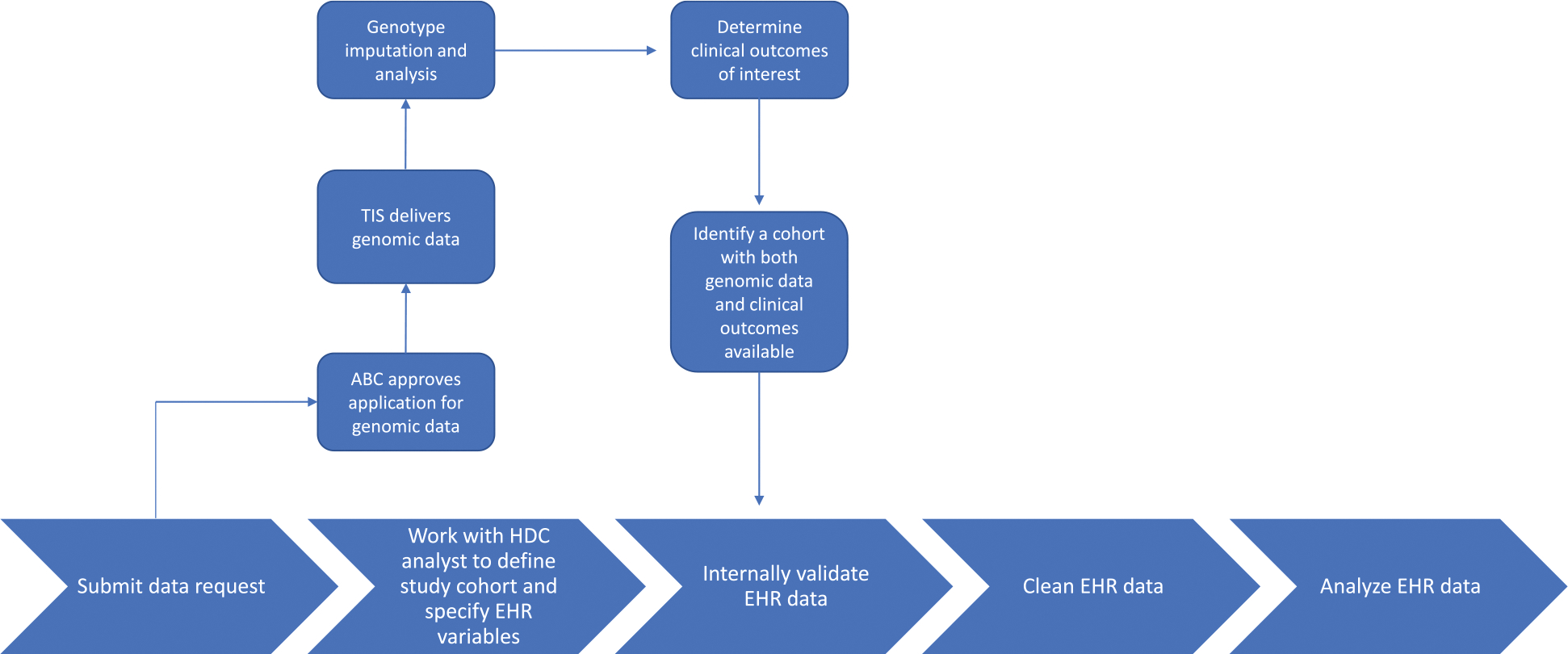
Process overview for working with EHR data warehouse. ABC, Access to Biobank Committee; TIS, Translational Informatics Services; HDC, health data compass; EHR, electronic health record

Finally, the cleaned dataset can be linked with additional data sources, such as metabolomic or genomic data. The amount of server space required to store all files for ‘omics-based precision medicine studies can be very large. For example, a whole genome survey (WGS) file for one subject is at least 30 GB and a raw metabolomics dataset can be over 60 GB. These server space requirements should be anticipated in preparation for this stage of the study. The processes for integrating data from our data warehouse with the Biobank at the Colorado Center for Personalized Medicine (CCPM Biobank) have been detailed elsewhere [9]. Figure 1 provides an overview.

#### Utilization of genomic data

The logistics for genomic data delivery from CCPM are specific to the data available from Translational Informatics Services (TIS). These data are mapped to HDC by either medical record number (MRN), which matches to the person ID, or contact serial number (CSN), which matches to the encounter ID. As expected, the initial data acquisition steps remain the same with regard to meeting with the HDC analyst, defining variables of interest, and working through the data request process.

More than 180,000 people have consented to participate in the CCPM biobank and more than 60,000 participants have been genotyped on the Illumina Infinium MEGA-EX chip. The investigators and faculty at the University of Colorado provided input regarding which single nucleotide polymorphisms (SNPs) are of greatest research interest with a significant focus on SNPs with known or suspected pharmacogenomic associations. The version of the Illumina Infinium MEGA-EX chip used to genotype CCPM biobank participants includes many of the SNPs they selected. This chip genotypes ~ 2.2 million SNPs with the ability to impute ~ 50 million SNPs.

The Access to Biobank Committee (ABC) reviews applications for use of the CCPM biobank. The investigator must complete a study proposal form specific to the ABC to receive consideration for study approval. It is worth noting that the IRB protocol may need to be revised if using genotyping data, which is considered PHI. As with the IRB submission, the ABC may require revisions to the study protocol or additional clarifications prior to granting access to biological specimens, genotyping data, PHI, and recontact of participants. After obtaining approval, the investigator must decide how to receive genomic data. This will depend primarily on available storage space and analytical capabilities.

TIS delivers genotyping data in one of four ways depending on the investigator’s preference and experience. Each of these delivery methods has advantages and potential drawbacks. In the first, CCPM delivers non-imputed genotype data from the Illumina chip. This method requires significant time and effort on the part of the investigator, who must either possess or have access to the requisite expertise, but also offers flexibility in approach to statistical analyses.

In the second, TIS delivers genotype data that is already imputed and has undergone quality control. This method may require significant storage space but the format is more accessible. TIS provides imputation rates with the data delivery such that the investigator can interrogate the data as appropriate, but the investigator does not have access to the original raw genotype dataset.

A third option is that TIS delivers genotype data for a prespecified region of a gene or list of SNPs. These data are typically derived from prior WGS or genome wide association studies (GWAS). This data delivery method is relatively expeditious and most appropriate for candidate gene studies or validation studies. TIS staff are available to confirm which genes and SNPs have sufficient coverage on the MEGA-EX chip. This resource helps ensure that previously identified genomic associations are not excluded in the analyses.

Finally, as a fourth option, TIS completes all imputation, quality control, regression analyses, and basic manuscript figure development. As a prerequisite, the investigator must provide the arbitrary identification numbers assigned by HDC, the outcome variables, and the study covariates. This data delivery method demands the fewest resources and the least amount of expertise from the investigator. Building the genotype and imputation pipeline takes 1–3 months as outcome variables are well defined and ancestry principle component analyses are validated. For established genomic pipelines, principle component analyses are relatively static, saving time for developing the genomic analysis pipeline. Following pipeline refinement, TIS provides the delivery via the server OneDrive, and the turnaround time after submission of the requisite variables is approximately 1 to 2 weeks.

### Challenges of using data warehouses for research

EHRs are designed for clinical care, and there are challenges to consider when repurposing EHR data for research. These challenges stem from imperfect and fragmented data as well as patient privacy and security protections.

#### Variability in EHR data format and quality

EHR data include both structured and unstructured data. Structured data consist of discrete variables that capture controlled vocabulary. Common examples include laboratory values, medications, allergies, immunizations, vital signs, and encounter diagnoses. Unstructured data comprise free-text, narrative notes in the medical record. EHR data are complex and structured and unstructured data may overlap. For example, flowsheets used to record vital signs may include free-text descriptors.

Both data types have advantages and drawbacks. Structured data reduce ambiguity but limit expressivity. They are relatively easy to retrieve, can reduce the time required for coding, and can streamline data analysis. Unstructured data provide complex and detailed clinical information, but with a spectrum of quality and completeness in documentation [10]. Natural language processing (NLP) is a form of machine learning that scans narrative data to extract computable results for analysis. Many NLP programs are currently available, although limited in ability to provide sufficiently detailed analysis to appreciate linguistic nuances and clinical concepts [2].

#### Defining clinical Phenotypes from EHR data

Several methodological approaches are available to define clinical phenotypes in EHRs. These approaches include manual chart review, rule-based phenotyping, machine learning, and phenome-wide approaches (PheWAS) [11, 12]. Each of these strategies requires a range of clinical and informatics expertise, and none comprehensively captures the information available in EHRs [11, 12]. Since accurate phenotyping is crucial for correct identification of study cohorts, further refinement of phenotyping algorithms will inform rigorous EHR-based research.

Clinical phenotyping algorithms facilitate the extraction of clinical data from diverse EHRs in a consistent manner. The Electronic Medical Records and Genomics (eMERGE) Network is a multisite network of health systems that combines DNA biorepositories with EHRs. eMERGE applies electronic algorithms to investigate phenotypic associations with genetic variants [13]. The network highlights the importance of accurate phenotyping and the potential for these methods to accelerate discovery research.

#### Accuracy of EHR data

Although EHRs contain vast amounts of data for clinical phenotyping, the utility of EHR data for research purposes is limited by EHR inaccuracy [8]. Common sources of EHR inaccuracy include diagnostic uncertainty, billing errors, and incomplete documentation. Diagnoses evolve over time, and a clinician may initially bill for a suspected diagnosis that is later deemed incorrect. A clinician may enter the wrong billing code or upcode diagnoses for higher reimbursement. Billing limited to a certain number of codes per visit often biases toward codes with the highest reimbursement such that “cheaper” codes are underrepresented in the medical record. Furthermore, clinical data are often fragmented since EHRs are not centralized. Patients may utilize multiple healthcare systems depending on insurance status and geography, and this can result in missed cases [2].

#### Missing data

Missing data are a common problem in all areas of medical research, including EHR-based studies. The investigator should anticipate that data will be missing and have a method to address this as part of the study’s analytic plan. Missing values often do not occur at random, such that the amount of missing data and subject characteristics associated with missingness should be identified. Although subjects with missing data could be excluded from the analysis, this approach results in bias and loss of power. A better approach is to perform multiple imputation of missing values. Multiple imputation is a rigorous statistical technique that replaces each missing value with a plausible value based upon known characteristics of the dataset [14–17]. This allows for the inclusion of all subjects in the analysis, including those that would have been excluded due to missing values, and a less biased and more precise estimate of the outcomes under study [15, 17, 18].

#### Lack of discrete validated clinical outcome elements

Many EHRs, including the EHR associated with the University of Colorado Health System, do not routinely include discrete tools for common outcomes. Validated clinical tools (e.g., HEART score for cardiac events, PHQ-9 for depression severity) would be helpful if incorporated into the medical record. Investigators could use scores from these tools, if routinely captured, to more efficiently collect and assess retrospective data.

#### Privacy and security

EHR-linked biobanks pose privacy and security concerns. They have historically operated under various consent models, with the traditional gold standard of informed consent considered problematic for four reasons: (1) Informed consent considers only the individual and does not account for close relatives; (2) Consent cannot be “informed” at the time it is obtained since future research on those samples is unknown; (3) Biobanks are a resource for a vast number of research projects such that obtaining informed consent for each project is impossible; and (4) It is unlikely that an individual’s right to later withdraw consent can be fully respected [19]. The NIH’s Genomic Data Sharing policy, which went into effect January 25, 2015, requires patients to consent to sharing of their DNA and made many existing opt-out consent models untenable [2, 20]. EHR-linked biobanks are associated with various institutions and currently fragmented. Linking this data is desirable for large-scale research studies, although data become more difficult to deidentify with increasing quantities of linked data [21].

## Conclusions

In summary, this review considers the advantages, potential drawbacks, and approaches to utilize EHR data for secondary research. We discuss methods to optimize efficiency in obtaining large amounts of data while protecting patient privacy, methodological and statistical considerations to maintain data integrity, integration with additional data sources, and ongoing privacy and security concerns.

Health data warehouses have tremendous potential to support research and discovery. Repurposing EHR data for research is an iterative process that requires familiarity with institutional resources and process requirements. Advances in EHR data abstraction, integration with biobanks, and linkage across institutions are essential to fully realize the potential of health data warehouses for clinical research. Furthermore, inter-institutional collaboration and harmonization are requisite to support the translation of EHR-based research to inform clinical care.

## Data Availability

Not applicable.

## Abbreviations

HER: Electronic health record
IRB: Institutional review board
HDC: Health Data Compass
PHI: Personal health information
IT: Information technology
